# MBLEformer: Multi-Scale Bidirectional Lesion Enhancement Transformer for Cervical Cancer Image Segmentation

**DOI:** 10.2174/0115734056357180250516022218

**Published:** 2025-09-16

**Authors:** Shuhui Li, Peng Chen, Jun Zhang, Bing Wang

**Affiliations:** 1 Anhui University, Institutes of Physical Science and Information Technology & School of Internet, Information Materials and Intelligent Sensing Laboratory of Anhui Province, National Engineering Research Center for Agro-Ecological Big Data Analysis & Application, Hefei 230601, China; 2 Anhui University of Finance & Economics, School of Management Science and Engineering, No. 962 Caoshan Road, Bengbu 233030, Anhui, China

**Keywords:** Cervical cancer, Lugol's Iodine Staining, Image segmentation, Transformer, Attention mechanism, upsampling

## Abstract

**Background:**

Accurate segmentation of lesion areas from Lugol's Iodine Staining images is crucial for screening pre-cancerous cervical lesions. However, in underdeveloped regions lacking skilled clinicians, this method may lead to misdiagnosis and missed diagnoses. In recent years, deep learning methods have been widely applied to assist in medical image segmentation.

**Objective:**

This study aims to improve the accuracy of cervical cancer lesion segmentation by addressing the limitations of Convolutional Neural Networks (CNNs) and attention mechanisms in capturing global features and refining upsampling details.

**Methods:**

This paper presents a Multi-Scale Bidirectional Lesion Enhancement Network, named MBLEformer, which employs the Swin Transformer encoder to extract image features at multiple stages and utilizes a multi-scale attention mechanism to capture semantic features from different perspectives. Additionally, a bidirectional lesion enhancement upsampling strategy is introduced to refine the edge details of lesion areas.

**Results:**

Experimental results demonstrate that the proposed model exhibits superior segmentation performance on a proprietary cervical cancer colposcopic dataset, outperforming other medical image segmentation methods, with a mean Intersection over Union (mIoU) of 82.5%, accuracy, and specificity of 94.9% and 83.6%.

**Conclusion:**

MBLEformer significantly improves the accuracy of lesion segmentation in iodine-stained cervical cancer images, with the potential to enhance the efficiency and accuracy of pre-cancerous lesion diagnosis and help address the issue of imbalanced medical resources.

## 1 INTRODUCTION

Cervical cancer represents one of the most prevalent gynecological malignancies, with a significant impact on women's health, characterized by high incidence and mortality rates [1]. Notably, the distribution of cervical cancer cases is highly uneven. In 2022, approximately 94% of the roughly 350,000 deaths from cervical cancer occurred in low- and middle-income countries.

While there has been a decline in the incidence of cervical cancer is declining in some developed countries, there has been an increase in mortality rates on an annual basis in underdeveloped regions [2]. Fortunately, cervical cancer has a relatively long pre-cancerous lesion period, and early detection and treatment can achieve a cure rate of over 90% [3]. It is therefore imperative that cervical cancer screening be conducted regularly to prevent the disease from progressing.

In economically developed regions, the Pap smear test is the most common method used for the prevention and control of cervical cancer. Patients who test positive undergo further colposcopic examination and biopsy. Those with high-grade squamous intraepithelial lesions are treated under the relevant guidelines [4]. However, in many resource-limited areas, cytological diagnostic systems remain underdeveloped, thereby impeding the advancement of screening. Consequently, colposcopic iodine staining tests are frequently the preferred method for cervical cancer screening in underdeveloped regions [5]. However, manual delineation of the lesion areas requires a high level of expertise and focus from pathologists, which is often limited by the scarcity of experienced medical personnel. Moreover, pathologists' diagnoses are influenced by subjectivity and personal experience, leading to inevitable errors in image annotation, which in turn increases the risk of misdiagnosis and missed diagnoses. Automated deep learning techniques can effectively assist pathologists in improving the efficiency and accuracy of early cervical cancer diagnosis, thereby significantly reducing the likelihood of misdiagnosis. Consequently, the advancement of computer-aided techniques to achieve automated segmentation of lesion regions in colposcopic iodine-stained images has become a pressing necessity. With the advancement of computer-aided image automatic segmentation technology, deep learning methods have facilitated significant advancements in the medical field [6], reducing resource and labor consumption compared to traditional methods.

In recent years, several CNN-based models have been developed for cervical cancer image segmentation [7, 8]. However, due to their fixed receptive fields and relatively simple structures, they often encounter difficulties in accurately extracting appearance features when dealing with structurally simple yet morphologically variable cervical images, which can result in overfitting. Conversely, the advancement of Transformers in the field of computer vision has been both rapid and pervasive. For instance, the Vision Transformer (ViT), as proposed by Dosovitskiy* et al.* [9], and the Swin Transformer, as proposed by Liu *et al*. [10], have attracted considerable interest from researchers engaged in the field of medical image applications. An increasing number of researchers are employing suitable Transformer-based segmentation methods or modifying Transformer structures to enhance efficiency in addressing medical issues. Although Transformers are highly adept at processing global contextual information, their segmentation performance still exhibits potential for improvement due to a lack of spatial bias. This is particularly evident in tasks that necessitate the detailed modelling of local information, such as medical image segmentation.

To address these issues, this paper proposes a network model based on Swin Transformer, a multi-scale attention mechanism, and a bidirectional lesion enhancement module. This model is called the Multi-Scale Bidirectional Lesion Enhancement Transformer (MBLEformer). The model extracts feature maps at three distinct stages through the use of a Swin Transformer encoder, thereby enabling the network to learn feature information with greater depth. In light of the intricate nature of lesion distribution and area segmentation in medical images, this paper proposes the incorporation of a multi-scale attention module, which extracts features from four distinct scales. This module employs convolution kernels of varying sizes to convolve the three-stage feature maps, thereby enhancing the network's capacity to acquire multi-scale feature information and addressing the issue of channel attention neglecting positional information. To achieve a comprehensive focus on both local and global information, this paper introduces a bidirectional lesion enhancement upsampling module in the decoder part, which is designed to further refine the edge structure and detail information of the images, thereby improving the predictive accuracy of the network model. The proposed MBLEformer was evaluated on our proprietary cervical cancer colposcopic dataset and compared with several advanced methods. The experimental results demonstrate the superiority of the proposed method. Additionally, adaptive parameter and ablation studies of each module were conducted, proving the effectiveness of the proposed network modules in the task of cervical cancer iodine-stained image segmentation.

The primary contributions of this paper are as follows:

(1) The integration of Swin Transformer into the task of cervical cancer iodine-stained image segmentation has been shown to enhance the model's learning and generalization capabilities, thereby achieving higher segmentation accuracy.

(2) The design of a multi-scale attention mechanism model, which comprehends lesion regions in images from different convolutional perspectives, enhances the network's ability to learn and extract various morphological features in iodine-stained cervical cancer images.

(3) The proposal of an adaptive bidirectional lesion enhancement upsampling strategy that refines lesion area features from both forward and backward angles.

## 2 RELATED WORK

This section presents an overview of traditional cervical cancer screening methods and the evolution of deep learning techniques for medical image segmentation. Furthermore, this section provides an overview of the advancements in research on Transformer and attention mechanisms.

### 2.1 Current Status of Traditional Cervical Cancer Screening Methods

The traditional methods for early cervical cancer screening include: (1) Visual inspection methods, including acetic acid white staining and Lugol's iodine staining of the cervix; (2) Pap smear, where cells are collected from the squamocolumnar junction of the cervix and examined under an optical microscope for the presence of abnormal cells; (3) Thin-layer liquid-based cytology, where involving the collection of cervical cells in a liquid medium to remove impurities, followed by centrifugation, slide preparation, and staining to obtain high-quality, clear cervical cell samples for determination of their benign or malignant nature [11, 12]. In comparison to the latter two cytology-based screening methods, the colposcopic Lugol's iodine staining method for cervical cancer screening offers several advantages, including the fact that it is non-invasive and low-cost. Furthermore, the majority of hospitals possess colposcopes, rendering this cervical cancer screening method a preferred option in resource-limited regions [13]. It is important to note that the results of these screening methods serve merely as reference indicators, necessitating a subsequent biopsy of the patient's lesion areas to ascertain the stage of the pre-cancerous lesions with certainty [14].

The growth in the quantity of pathological image data about cervical cancer has been exponential with the advent of sophisticated medical imaging technology. In comparison to small data from a limited number of samples, a substantial quantity of pathological images can facilitate the discovery of a greater range of potential information and patterns. However, traditional pathological diagnosis of cervical cancer is limited by factors such as low automation and prolonged diagnostic times [15]. Fortunately, the abundance of data resources currently available, coupled with the rapid advancement of computing power, has enabled artificial intelligence technology to achieve remarkable results in the field of image recognition. In recent years, deep learning methods have been widely applied to assist in cervical cancer screening. For instance, neural networks are used to classify, segment, and detect targets in cervical cancer pathological images, aiding medical diagnosis. To some extent, these methods have alleviated the diagnostic pressure on pathologists and have filled the gap caused by the lack of experienced doctors in resource-poor areas.

### 2.2 Medical Image Segmentation

In recent years, CNNs have been widely applied in medical image segmentation, achieving notable success in the segmentation of cervical images and in assisting in the diagnosis of medical conditions. Ronneberger *et al*. proposed the U-Net model, which was one of the first networks to be applied to biomedical image segmentation [16]. Since its introduction in 2015, the U-Net model has been widely used in various medical image segmentation tasks, inspiring numerous improved models based on its architecture [17, 18]. Qian *et al*. proposed an image segmentation method based on an improved fully convolutional network [19], which incorporates dilated convolutions at four different rates to capture multi-scale contextual feature information, thereby enhancing the accuracy of target contour extraction.

Xing *et al*. proposed SegMamba [20], a 3D medical image segmentation method that integrates the Mamba state space model. This method captures long-range dependencies while maintaining lower computational costs compared to Transformer-based approaches. Jin *et al*. proposed the DASC-Net model [21], which is used for segmenting infection regions in COVID-19 CT images and effectively addresses the issues of limited datasets and domain shift. Wang *et al*. proposed the Mamba-UNet model [22], which combines the advantages of U-Net in medical image segmentation with the capabilities of the Mamba architecture, enabling the preservation of spatial information across different scales of the network, thereby achieving comprehensive feature learning. Ding *et al*. proposed a universal medical image segmentation framework, S2VNet [23], which leverages 2D networks and clustering propagation methods to reduce inference time and memory consumption, thereby enhancing segmentation efficiency.

#### 2.2.1 Current Research on Cervical Cancer Image Segmentation

In the context of cervical imaging, Meng *et al*. proposed a layered spatial pyramid network [24], wherein vertical and horizontal layered spatial pyramid structures were devised to aggregate multi-scale information during feature extraction, thereby enhancing the differentiation of lesion levels in cervical pre-cancerous lesion images. Liu *et al*. proposed a deep learning-based method for acetic white area segmentation in cervical images [25]. The ASPP module and feature integration structure in the DeepLab V3+ network were optimized through an improved global attention network. Elakkiya *et al*. introduced a cervical cancer diagnosis system based on a hybrid object detection adversarial network [26], which combines Faster R-CNN with GAN to automatically locate and segment pre-cancerous lesion areas in cervical images. Kim *et al*. proposed a convolutional neural network classification method based on acetic white epithelium image segmentation [27]. This method employs the SegNet network for image segmentation, to obtain acetic white areas before classification. The aim is to enhance the accuracy of the ResNet model in classifying cervical intraepithelial neoplasia.

#### 2.2.2 Research Status and Challenges of Iodine-Stained Image Segmentation

Iodine-stained images are an important tool for early cervical cancer screening, especially in resource-limited regions. Due to its low cost, minimal harm, and absence of corrosiveness like acetic acid, iodine staining has become one of the preferred methods for cervical cancer screening. By applying iodine staining to cervical tissue, doctors can segment the lesion areas in the image, thereby aiding in lesion detection.

In recent years, deep learning techniques have been applied to the automatic analysis of Lugol's iodine-stained images, aiming to improve the accuracy of screening and reduce the burden on doctors [ 28]. However, most AI tools currently learn only from acetic acid cervical images, and single-channel acetic acid cervigrams tend to exaggerate false-positive warnings. Ling Yan *et al*. proposed the HLDnet model [ 29], which can fuse acetic acid and Lugol iodine cervical images for segmentation, thus improving the accuracy of lesion detection more comprehensively. However, this method still shows low accuracy for single-channel detection when only acetic acid or Lugol iodine cervicograms are available.

Although deep learning methods have made some progress in the segmentation of Lugol iodine-stained images, several challenges remain. First, Lugol iodine-stained images have low contrast, making it difficult to distinguish between lesion areas and normal tissues. Additionally, lesions in iodine-stained images exhibit diverse shapes, presenting a significant challenge in training deep learning models. Traditional algorithms often perform poorly when handling subtle lesions or lesions with blurry boundaries [ 30]. Due to reliance on clinical experience and the low quality of data, current research on iodine-stained cervical images has mainly focused on classification tasks [ 31], with limited in-depth research and applications in segmentation.

Another challenge is the lack of annotated datasets for Lugol iodine-stained images, which makes deep learning models face the problem of insufficient data during training. Currently, deep learning-based segmentation methods are mainly used at the cervical cell level [ 32]. Therefore, there is an urgent need to develop more accurate and reliable iodine-staining image segmentation methods to improve the automation and accuracy of cervical cancer screening.

In conclusion, deep learning technology has been extensively utilized for the analysis of cervical cancer pre-cancerous lesions, achieving superior recognition accuracy compared to traditional machine learning algorithms. The remarkable success of deep learning methods in image segmentation highlights their potential for automatically segmenting lesion areas in colposcopic images of cervical cancer. However, applications specific to iodine-stained image segmentation remain relatively limited.

### 2.3 Application of Transformers

In this paper, “*Attention Is All You Need*” [33], Ashish Vaswani *et al*. introduced the Transformer model, which effectively handles long-distance dependencies through its multi-head attention mechanism, significantly enhancing the model's parallel processing capability and training efficiency. Dai *et al*. put forth the TransMed model [34], which fuses convolutional neural networks and Transformers and establishes long-range dependencies between different modalities, marking the inaugural application of Transformers in multi-modal medical image classification and enhancing classification accuracy. Chen *et al*. introduced the TransUNet network model [18], which combines Transformers with the U-Net architecture for medical image segmentation. This addresses the limitations of traditional U-Net in modeling long-range dependencies by introducing a global self-attention mechanism and cascade upsampling modules. Sun *et al*. incorporated Transformers and dual attention blocks into the conventional U-Net framework with their DA-TransUNet [17], effectively filtering superfluous information and enhancing the precision of medical image segmentation. Liu *et al*. proposed the Swin Transformer [10], which applies Transformers to vision tasks by introducing a hierarchical structure and shifting window technique. This effectively reduces computational complexity while achieving efficient global self-attention computation, significantly improving the performance of Transformers in image classification, object detection, and semantic segmentation tasks.

The CoTr model [ 35] proposed by Zhu *et al*. combines convolutional neural networks and the Transformer architecture for the automatic tumor segmentation and survival prediction in cervical cancer images. This model effectively captures the global contextual information in cervical cancer MRI images through the self-attention mechanism of the Transformer, which further improves the accuracy of tumor segmentation. Nirmala *et al*. [ 36] proposed a cervical cancer image segmentation method that combines an adaptive deep residual aggregation network and an adaptive visual Transformer encoder, achieving high-precision segmentation of different types of cervical cancer cells. Kim *et al*. [ 37] proposed a dual network method that integrates convolutional neural networks and Transformers, realizing automatic tissue segmentation in cervical cancer radiotherapy and improving treatment accuracy through real-time needle tracking.

Despite the success of Transformer-based methods in medical image segmentation, they tend to neglect positional and channel information in images. This results in a lack of capture of complex structures and detailed features in cervical images. Therefore, further optimization is necessary to accurately segment lesion areas.

### 2.4 Application of Attention Mechanisms

Attention mechanisms serve as the foundation and core components of Transformer models, representing a significant advancement in the implementation and expansion of attention mechanisms in deep learning.

Tang *et al*. proposed a joint attention block [38], which combines spatial and channel attention to determine the importance of each channel and generate spatial attention maps to supplement channel attention. This enables the network to learn richer feature representations during segmentation. Abboodi *et al*. introduced a high-resolution model based on deep UNet and multiple attention mechanisms [39-45], which significantly improved segmentation accuracy and clinical applicability by combining spatial and channel attention mechanisms.

Sornapudi *et al*. [ 40] proposed a cervical histology image classification model based on the attention mechanism, which effectively optimized the segmentation of key regions in cervical epithelial images by combining the feature extraction capabilities of CNN and RNN through the attention mechanism. Abboodi *et al*. [ 41] Introduced a dual attention mechanism (CBAM and CA), which not only enhanced the extraction capabilities of local and global features but also improved the localization accuracy of cervical lesion areas. An *et al*. [ 42] proposed a Mobile Attention block that, while capturing local features, also processes long-range global information, helping the model focus on the cellular features of different differentiation levels of cervical cancer. He *et al*. [ 43] Introduced the Shuffle Attention-based Refining Bottleneck module, combined with the Oblique Attention Connection Module, to effectively address the issues of edge blurring and detail loss in cervical cancer image segmentation tasks by refining features at different scales through diagonal attention connections. Liu *et al*. [ 44] integrated Squeeze-and-Excitation blocks and attention gates into the U-Net architecture, significantly improving the automatic segmentation of tumors, uterus, and vagina in cervical cancer images.

To more effectively process multi-scale feature information during segmentation and to enhance the frequently neglected local detail features in the process of segmenting cervical cancer lesions, further optimization of methods for accurately segmenting iodine-stained images of cervical cancer is necessary, under the specific requirements of this task.

## 3 METHOD

**Fig. (1):**
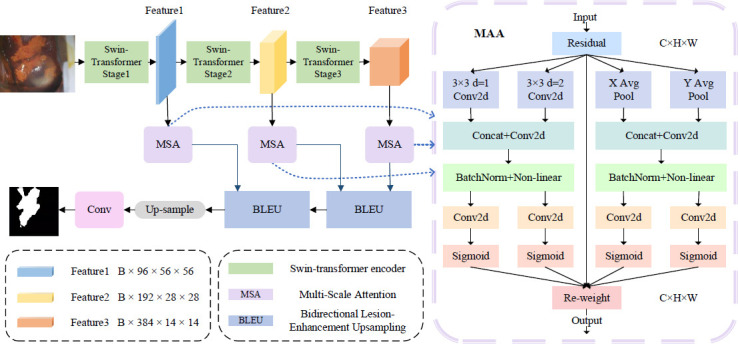
Overall Structure of MBLEformer. The Swin Transformer serves as the encoder, extracting multi-level local features. The MSA module extracts feature information from multiple scales. The BLEU is a bidirectional lesion enhancement upsampling module.

The overall structure of the network proposed in this paper is illustrated in Fig. (**1**). The MBLEformer is principally constituted by a Swin Transformer encoder with 16× downsampling, three multi-scale attention (MSA) modules, and two bidirectional lesion enhancement upsampling modules. The detailed architecture of the MSA module is illustrated within the dashed box on the right side of (Fig. **1**).

The backbone network of the proposed multi-scale bidirectional lesion enhancement cervical cancer image segmentation model employs the Swin Transformer encoder module. This structured visual Transformer is capable of progressively learning global feature information at varying scales, effectively addressing the challenge of feature capture in high-resolution image processing. Next, the multi-scale attention mechanism extracts image feature information at three different stages, reassigning weights to features of varying importance. Subsequently, in the decoder part, bidirectional lesion enhancement upsampling (BLEU) modules are employed to extract additional features, thereby refining and enhancing the representation of cervical cancer lesion features and consequently enhancing the accuracy of the network’s segmentation. The following section provides a detailed description of the structure of each component of the model.

### 3.1 Pyramid Swin Transformer

The proposed method employs a hierarchical Swin Transformer encoder as the backbone network, leveraging its shifted window mechanism to effectively capture global long-range dependencies [10]. This design addresses the limitations of traditional CNNs in handling long-range dependencies and global feature extraction. The Swin Transformer enhances its modeling capability by connecting information across windows using a sliding window approach.

When processing large-scale, high-resolution images, traditional CNNs often face the bottleneck of rapidly increasing computational complexity as the image size grows. In contrast, Swin Transformer mitigates this issue with its shifted window approach, performing self-attention computation within local windows. This results in linear computational complexity concerning the image size, making it highly efficient for segmenting high-resolution cervical lesion images.

Furthermore, Swin Transformer improves the global self-attention computation in traditional Transformers by progressively reducing the feature map resolution. This allows the network to layer-by-layer identify the relationships and interactions between image patches. This approach not only enhances the capture of global information but also refines the local details of lesion regions.

In conclusion, the Swin Transformer offers significant advantages in improving the segmentation accuracy and computational efficiency of cervical iodine-stained images, especially in resource-constrained environments, where it can substantially enhance diagnostic efficiency and accuracy.

### 3.2 Multi-Scale Attention Mechanism

In the context of cervical cancer iodine-stained image segmentation, it is notable that the lesion and non-lesion areas exhibit significant differences in size, shape, and color. Therefore, the use of a single convolution kernel for feature extraction presents a challenge for the network in handling multi-scale feature information, with the potential for local detail features to be overlooked during the segmentation. The conventional attention mechanisms are deficient in the representation of features.

We have devised a multi-scale attention mechanism that is distinct from both channel and spatial attention mechanisms. This mechanism is capable of discerning the lesion areas in the image from a multitude of convolutional perspectives. The detailed structure of the MSA module is illustrated in the diagram on the right-hand side of (Fig. **1**).

The multi-scale attention mechanism is capable of discerning the semantic relationships between the horizontal and vertical orientations of image features, as illustrated by the two branches on the right side of the MSA module diagram. Furthermore, it captures the semantic information of each pixel and its surrounding pixels using convolutions with varying dilation rates, as illustrated by the two branches on the left of the MSA module diagram. Ultimately, the feature information obtained from the four distinct scales is integrated to generate multi-scale attention weights that strike a balance between spatial and channel information, thereby providing a robust feature representation for the network's diverse components.

The specific implementation process is as follows:

The initial stage of the process entails the extraction of feature information at varying scales from the input feature map. Assuming the input is *X* ϵ *R*^*c*×*h*×*w*^For key pixel information, a convolution kernel with a size of k=3×3 and a dilation rate of d=1 is employed, whereas for information surrounding key pixels, a convolution kernel with a size of k=3×3 and a dilation rate of d=2 is utilized. Adaptive average pooling is then applied to convert horizontal and vertical information into one-dimensional features.

The second stage entails the concatenation of the information on the pixel points and their surrounding regions, as illustrated below (eq. **1**):

**Table:** 

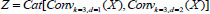	(1)

The data, Z, undergoes a series of processing steps, beginning with convolution and normalization operations, followed by non-linear activation, as illustrated in the following formula (eq. **2**):

**Table:** 

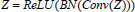	(2)

The processed features, Z, are then split into key pixel points, *Za=1*, surrounding pixels, *Za=2*,, using the Split function, and subsequently, importance feature maps are generated using convolution operations and the Sigmoid function, as illustrated below (eqs. **3**-**5**):

**Table:** 

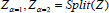	(3)

**Table:** 

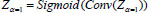	(4)

**Table:** 

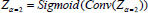	(5)

Similarly, the feature information in the horizontal and vertical directions is processed using a similar method. In this context, AvgPooLh denotes horizontal average pooling, and AvgPooLw denotes vertical average pooling, as illustrated below (eqs. **6**-**10**):

**Table:** 

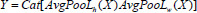	(6)

**Table:** 

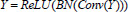	(7)

**Table:** 

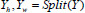	(8)

**Table:** 

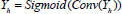	(9)

**Table:** 

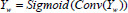	(10)

It is worth noting that when concatenating feature maps, their size is represented as C×H×W, whereas the concatenated feature map has a size of 2C×H×W. After convolution and normalization, only the channel dimension is altered, with the spatial dimensions remaining unchanged.

In the third stage, the important feature maps derived from the four distinct perspectives (key pixel points, *Za=1*, surrounding pixels, *Za=2*, horizontal direction, *Yh*, and vertical direction, *Yw*) are integrated to re-assign weights to the input feature map, as illustrated below (eq. **11**):

**Table:** 

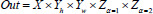	(11)

In the overall network structure, the three-stage feature maps extracted by the Swin Transformer are processed using this attention mechanism. The MSA module enables the network to capture positional information and channel relationships, facilitating multi-scale feature fusion and weight allocation for feature maps obtained from disparate receptive fields. This further augments the model's capacity to capture and segment features in intricate medical images. This attention mechanism effectively enhances the network's global feature extraction capability while preserving precise positional information, rendering it well-suited for the complex lesion areas in cervical cancer iodine-stained images.

### 3.3 Bidirectional Lesion Enhancement Upsampling Module

The prevailing approach to existing network upsampling involves the concatenation and convolution of downsampled features. However, this methodology fails to recognize that the essence of upsampling is to further refine the representational details of the prediction feature map based on the downsampled features. In light of the aforementioned considerations, this paper proposes a BLEU module, which is designed to enhance the details of lesion areas and their edges from both forward and backward perspectives. This approach enhances the distinction between target and irrelevant regions, thereby improving the accuracy of the network model's predictions. The detailed structure of the BLEU module is illustrated in Fig. (**2**).

Let us consider two sampled feature maps, *X1, X2*, with different resolutions as input. If the size of the feature map *X1* is twice that of the feature map *X2*, an upsampling operation is first performed on *X2* to enlarge it to the same size as *X1* before feature fusion.

**Fig. (2):**
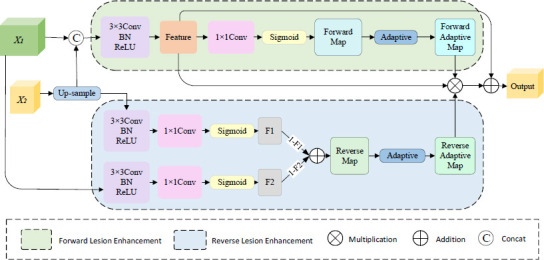
Bidirectional Lesion Enhancement Upsampling Module.

(1) Forward Lesion Enhancement:

As illustrated in the Forward Lesion Enhancement part of (Fig. **2**), the features of the lesion area are enhanced to improve segmentation accuracy using the following approach:

The initial step involves convolving the respective features of the two feature maps, which yields the initial importance map, Feature, of the target area as below (eq. **12**):

**Table:** 

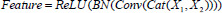	(12)

Next, the feature map is normalized using the Sigmoid function, thereby generating a weighted feature map for the lesion area (eq. **13**).

**Table:** 

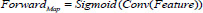	(13)

Then, an adaptive weighting method is employed whereby regions with weights exceeding or equaling α are assigned a value of 1, while regions with weights less than β are assigned a value of 0. Through binarization, the key focus area map of the lesion regions in the image is obtained (eqs. **14**-**15**):

**Table:** 

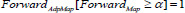	(14)

**Table:** 

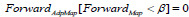	(15)

(2) Reverse Lesion Enhancement:

As illustrated in the Reverse Lesion Enhancement part of (Fig. **2**), inverse operations are performed on the two feature maps and extract features from the results of these inverse calculations to further enhance the characteristics of the lesion areas.

In particular, each feature map is subjected to a distinct convolutional feature extraction operation, thereby refining its detailed features as below (eqs. **16**-**17**):

**Table:** 

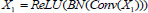	(16)

**Table:** 

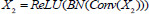	(17)

Next, the Sigmoid function is employed to ascertain the distribution of the regions of interest (eqs. **18**-**19**):

**Table:** 

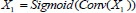	(18)

**Table:** 

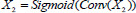	(19)

Then, the target area is segmented from a reverse perspective by subtracting the feature distributions of the two feature maps from 1, respectively. The resulting distributions are then summed to generate a reverse feature map, which serves to further refine the characteristics of the target area (eqs. **20**-**22**):

**Table:** 

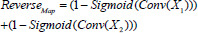	(20)

**Table:** 

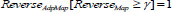	(21)

**Table:** 

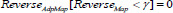	(22)

Once the forward-enhanced lesion area feature map and the reverse-refined lesion area feature map have been obtained, the initial target area importance map is multiplied by these maps and weighted. The incorporation of residual connections facilitates the reintegration of the feature representation, thereby enhancing the representation of the target area. This process amplifies the differences between the cervical lesion areas and the background image, effectively handling the complex boundaries in iodine-stained images (eq. **23**):

**Table:** 

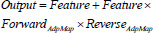	(23)

This marks the conclusion of one cycle of the bidirectional lesion enhancement upsampling operation. The resulting feature map, now classified as a low-resolution upsampled feature, will be integrated with the high-resolution downsampled features in the subsequent cycle of processing.

## 4 DATASET

The dataset utilized for training and testing the network model in this paper was obtained from hospitals and comprises records from 2,250 patients. Each record contains the information pertaining to a single patient and includes the patient's original colposcopic images, images obtained after application of acetic acid reaction, images obtained after iodine staining, and diagnostic reports from several professional physicians. The original images utilized in this experiment are divided into two distinct categories: the first category comprises 2,250 colposcopic images of the cervix following iodine application, while the second category encompasses manually segmented images of cervical cancer lesion areas, delineated by professional physicians, which serve as the reference standard for evaluating the experimental outcomes of the model. The conventional visual inspection methodology for Lugol's iodine-stained cervical cancer images is based on the premise that the application of a 5% Lugol's iodine solution to the cervix surface results in the normal cervical epithelium appearing brown or black, while areas with diminished iodine absorption exhibit a mustard yellow or remain unstained, indicative of lesion areas [14]. A preliminary diagnosis can be made regarding the presence of disease in a given area based on the varying absorption levels of iodine observed in different parts of the cervix after iodine staining. The total dataset of iodine-stained cervical cancer images obtained consists of 2,250 images. The dataset was randomly split in a 9:1 ratio during the experiments, resulting in 2,025 images assigned to the training set and 225 images assigned to the test set used for evaluating the network model.

## 5 RESULTS AND DISCUSSIONS

### 5.1 Evaluation Metrics

To ensure an accurate and objective comparison of the predictive capability, learning ability, and other performance aspects of different networks, we rigorously controlled variables during the experiments. All networks were tested under identical conditions, utilizing the same dataset, learning rate, and optimization methods. The segmentation experiments yielded seven different evaluation metrics, with the expert-manually segmented cervical regions serving as the gold standard. The primary metric employed for the evaluation of the segmentation performance of the networks was the Mean Intersection over Union (MIoU). This metric represents the mean degree of overlap between the lesion areas predicted by the networks and the actual lesion areas. The calculation formula is as follows (eq. **24**):

**Table:** 

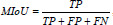	(24)

Where TP represents true positives, FP represents false positives, and FN represents false negatives. Additional performance evaluations are provided by other metrics. The recall metric indicates the degree of overlap between the correctly predicted lesion areas and the actual lesion areas identified by the model (eq. **25**).

**Table:** 

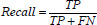	(25)

Specificity denotes the model's capacity to accurately identify and exclude non-lesion areas, where TN signifies true negatives (eq. **26**):

**Table:** 

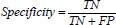	(26)

Precision denotes the proportion of pixels identified by the model as lesions that are true lesion areas (eq. **27**).

**Table:** 

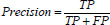	(27)

The F1 Score is a statistical measure that represents the harmonic mean of precision and recall. It is utilized to provide a comprehensive evaluation of the model's performance (eq. **28**).

**Table:** 

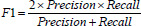	(28)

The F2 Score places greater emphasis on recall, increasing its weight to more accurately identify lesion areas (eq. **29**).

**Table:** 

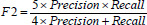	(29)

ACC_overall represents the overall accuracy, which is defined as the proportion of correctly predicted samples out of the total samples.

The F2 Score places greater emphasis on recall, increasing its weight to more accurately identify lesion areas (eq. **30**).

**Table:** 

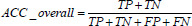	(30)

These metrics provide a comprehensive evaluation of the segmentation performance of each network model from multiple perspectives, including the ability to recognize positive and negative samples, detection precision, and the accuracy of the final segmentation results. By calculating and comparing the scores of existing methods on these metrics, it is possible to especially the mIoU metric, we can effectively validate the superiority and effectiveness of the proposed method in segmentation tasks.

### 5.2 Implementation Details

The experimental model presented in this paper was developed using the Python programming language, based on the PyTorch framework. The experiments were conducted on a Titan X GPU. The dimensions of the input images were set to 224×224 pixels. To enhance the model's generalization ability and robustness, data augmentation methods were employed, including random rotation, random horizontal flip, random vertical flip, and coarse dropout, with a probability of 0.3 for each. The optimizer utilized was the Adam optimizer, with an initial learning rate set to 1e−4. The learning rate was reduced by 0.1 throughout 40 epochs. The batch size was set to eight, and all experiments were trained for 150 epochs.

To optimize the model's performance in the cervical cancer iodine staining image segmentation task, this study utilized the Dice BCE Loss, a loss function that combines the advantages of Dice Loss and Binary Cross-Entropy (BCE) Loss.

The formula for Dice Loss is (eq. **31**):

**Table:** 

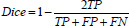	(31)

The formula for BCE Loss is (eq. **32**):

**Table:** 

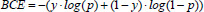	(32)

Here, y represents the ground truth label (0 or 1), and p is the predicted probability (ranging between 0 and 1).

BCE Loss exhibits good stability in the early stages of training, helping to accelerate model convergence. On the other hand, Dice Loss places greater emphasis on the similarity of the segmentation results, ensuring that the model generates smoother and more continuous segmentation boundaries when segmenting complex cervical cancer lesion areas. The Dice BCE Loss combines the stability of BCE Loss with the segmentation accuracy of Dice Loss, significantly improving the performance of iodine staining image segmentation.

### 5.3 Comparative experimental results

Fig. (**3**) illustrates the evolution of loss values throughout the training process of the proposed MBLEformer network. The horizontal axis represents the number of training epochs, and the vertical axis represents the loss values. As illustrated in the figure, our model rapidly reduces the loss value during the initial stage, indicating its capacity to learn effective features in a relatively short time and enhance segmentation efficiency. With the increase in training epochs, the loss value decreases steadily and tends to stabilize at a low value, which suggests good convergence of the model, a low risk of overfitting, and its suitability for handling complex cervical cancer iodine-stained images.

A self-constructed dataset was employed to evaluate the efficacy of the proposed network, with results compared to those of several established networks and state-of-the-art methods. The specific networks that were compared include the Unet [16], Unet++ [46], ResUnet++ [47], ViT_seg [38], Dual A_Net [48], CFANet [49], and Msraformer [39].

As demonstrated in Table **1**, our model demonstrated superior performance in segmenting cervical cancer iodine-stained images, achieving an mIoU of 82.5%, an overall accuracy of 94.9%, a specificity of 96.6%, and a precision of 83.6% on the test dataset. These evaluation results represent the most favorable outcomes among the networks under comparison. The proposed model demonstrates superior performance in most segmentation metrics when compared to other network models. The Recall was enhanced by 10.5% in comparison to the DualA_Net network, the Specificity improved by 1.4% in comparison to the ViT_seg network, the Precision improved by 5.0% in comparison to the Msraformer network, and the mIoU improved by 18.9% in comparison to the CFANet network. This illustrates that the network exhibits superior learning and predictive capabilities compared to the majority of existing models for the task of segmenting cervical cancer iodine-stained images.

Fig. (**4**) illustrates the evolution of the mIoU values of the network models throughout the training process in the comparative experiments. The horizontal axis represents the number of training epochs, and the vertical axis represents the mIoU values. As illustrated in the figure, with the increase in training epochs, the mIoU values of all models gradually increase and tend to stabilize. Our model consistently exhibits higher mIoU values across all epochs, especially in the later stages of training, significantly outperforming other comparison models. This indicates its superior performance in the task of segmenting cervical cancer iodine-stained images.

Fig. (**5**) illustrates a comparison of the computational speed for different networks, with measurements taken in seconds. The horizontal axis represents the names of different models, and the vertical axis represents the computation time required by each model to complete one iteration. As illustrated in the figure, our proposed model also demonstrates notable advantages in computational speed, necessitating only 134 seconds to complete one epoch, which is considerably less than the time consumed by other models. This indicates that our model not only outperforms other models in accuracy but also significantly reduces computational costs.

### 5.4 Ablation Study Results

To assess the efficacy of each module in the proposed network model, a series of ablation experiments and supplementary evaluations were conducted on the original dataset while maintaining the remaining network structure unchanged. The specific experimental setup and results are presented in Table **2**.

The initial row of Table **2** employs the fundamental structure of the original Swin Transformer model, retaining 32× downsampling as the baseline model for the purpose of assessing its performance on the given dataset. However, the results across various metrics were generally average.

To further optimize computational complexity while retaining detailed features, the second row of the table shows how the Swin Transformer model backbone was modified to 16× downsampling. The results demonstrated improvements across all metrics, with the mIoU increasing from 78.25% to 80.91%. The results of the ablation experiment demonstrate that 16× downsampling yields superior segmentation results than 32× downsampling. This is because it preserves spatial detail information more effectively and reduces computational complexity, which makes it a more suitable choice for our network model.

In the third row, we incorporated a multi-scale attention mechanism on top of the 16× downsampling. The results demonstrated further improvements in all metrics, indicating that the incorporation of the multi-scale attention mechanism has a beneficial impact on feature capture and segmentation accuracy.

In the fourth row of the ablation experiment, we further included the adaptive upsampling module based on the previous setup. The model achieved the best metrics, with mIoU increasing to 82.57%. The comparison indicates that the adaptive upsampling module significantly enhances boundary refinement and overall segmentation performance.

To identify suitable adaptive parameters for improving the segmentation performance of the bidirectional lesion enhancement upsampling module, we conducted ablation experiments by varying the hyperparameters α, β and γ while maintaining the parameters of other modules at their original values. As illustrated in Table **3**, the configuration with α=0.6, β=0.1, and γ=1.2 demonstrated the highest mIoU of 82.57%. This suggests that, under this configuration, the enhancement effect for cervical cancer lesion areas is optimal, and the model's segmentation performance is superior. These parameters were ultimately selected as the final model hyperparameters.

### 5.5 Result Visualization

Fig. (**6**) illustrates five examples of cervical cancer iodine-stained image segmentation results. The initial column depicts the original colposcopic images of the patients' cervices following the application of a 5% iodine solution. The subsequent grayscale images illustrate the segmentation outcomes derived from disparate network models. The dimensions of the image are consistent with those of the original colposcopic images. In the label images, the white regions with pixel values of 255 represent the unstained lesion areas in the colposcopic image post-iodine staining, whereas the black regions with pixel values of 0 represent the background and unstained normal areas of the colposcopic image. The second column, designated “GT,” depicts the corresponding binary label images of the original images. The white regions in the GT images have been manually annotated by experts and serve as the reference standard for the lesion areas in the cervical cancer images. The third to tenth columns display the visualization of the segmentation results of cervical cancer images obtained by different segmentation networks, with the rightmost column showing the segmentation results from the proposed network.

A comparison of the results reveals that our method produces superior segmentation outcomes. It accurately delineates the clearly defined, smoothly bounded lesion areas while preserving details, resulting in segmentation outcomes that are more closely aligned with the ground truth. For instance, in the initial row of segmentation outcomes, our network demonstrates no substantial omission in comparison to other networks, nor does it exhibit the more obvious over-segmentation as observed in the second row with other models. Notably, in addressing intricate backgrounds and nuanced characteristics, such as in the segmentation of the third to fifth images, our network effectively captures pivotal features and distinguishes them from the background, providing more reliable auxiliary support for the early screening of cervical cancer.

### 5.6 Failure Cases and Challenging Scenarios Analysis

Although MBLEformer demonstrates superior robustness compared to other methods in most experiments, achieving an overall accuracy of 94.9% on the test set, the model still exhibits issues of over-segmentation and under-segmentation in certain specific scenarios. Several examples are presented for analysis.

Over-segmentation Example: For the second and third iodine-stained images in Fig. (**6**) , the contrast between the lesion areas and the background is relatively low, and there is background noise such as glare. As a result, the MBLEformer model shows a degree of over-segmentation in the lesion areas, leading to some normal regions being mistakenly identified as lesions. To reduce such errors, we plan to optimize the edge detection and background suppression modules in the future, enhancing the model's ability to distinguish between lesion areas and normal tissues.

Under-segmentation Example: For the fourth and fifth iodine-stained images in Fig. (**6**), although the MBLEformer model accurately identifies the lesion areas, the lesions are quite small, and the model fails to fully segment the narrowest boundaries of these regions. To improve segmentation performance for such small lesion areas, we plan to incorporate more multi-scale training and finer local feature enhancement techniques.

By analyzing the model's performance in these complex scenarios, we have identified potential limitations in the current MBLEformer model and provided important directions for future model optimization.

## CONCLUSION

In this study, we proposed a multi-scale bidirectional lesion enhancement segmentation network model for the automatic segmentation of cervical cancer iodine-stained images. In particular, the Swin Transformer was employed as the encoder to initially extract multi-scale features from the images. The incorporation of a multi-scale attention module enabled the extraction of features from four perspectives: pixel points, surrounding pixels, the horizontal direction, and the vertical direction. This approach enhanced the network's capacity to extract both global and local features. In the decoding stage, we incorporated a bidirectional lesion enhancement upsampling module, which refines the lesion area features through an adaptive weighting method, thereby further enhancing the model's prediction accuracy and segmentation performance for pathological images.

In the experimental section, the efficacy of the proposed method and each module was validated through a series of comparative and ablation experiments conducted on our proprietary cervical cancer colposcopic dataset. The experimental results demonstrated that, in comparison to other advanced segmentation models, the proposed method yielded notable improvements in multiple metrics, with an mIoU of 82.57%, an overall accuracy of 94.9%, and a specificity of 96.6%. Furthermore, by adaptively adjusting the hyperparameters of the upsampling module, we further optimized the model's segmentation performance.

The improvement in segmentation performance indicates the significant potential of the MBLEformer model as a supplementary tool for cervical cancer screening. This model can assist doctors in more quickly and accurately identifying the lesion areas in cervical cancer iodine-stained images, thereby reducing misdiagnosis and providing more timely treatment for patients. Enhanced segmentation accuracy makes early screening more effective in clinical practice, particularly in resource-limited areas, where it can help avoid missed diagnoses and improve the overall accuracy of screening.

In future work, we will continue to refine the model architecture and investigate effective technological solutions for the early diagnosis and treatment of cervical cancer, aiming to address the challenge of imbalanced healthcare resources. Furthermore, we plan to extend the application of this model to other medical image segmentation tasks and explore its deployment feasibility. This will include optimizing the model for lightweight and real-time processing, making it more suitable for clinical use and enabling effective computer-aided diagnosis for healthcare professionals.

## Figures and Tables

**Fig. (3):**
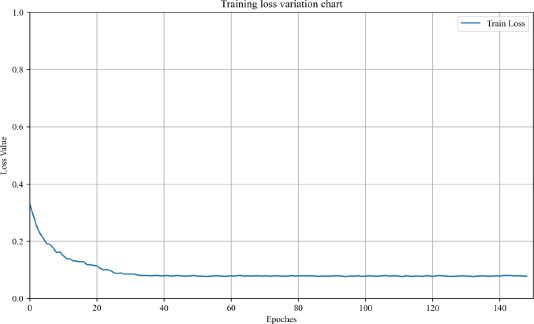
Change in Loss Values During the Training Process.

**Fig. (4):**
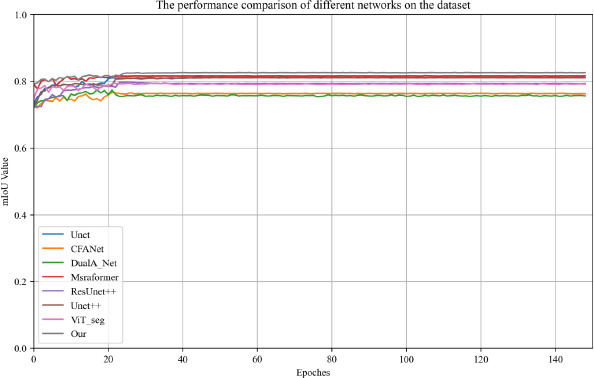
Comparison of the mIoU values during the training process for a variety of Networks.

**Fig. (5):**
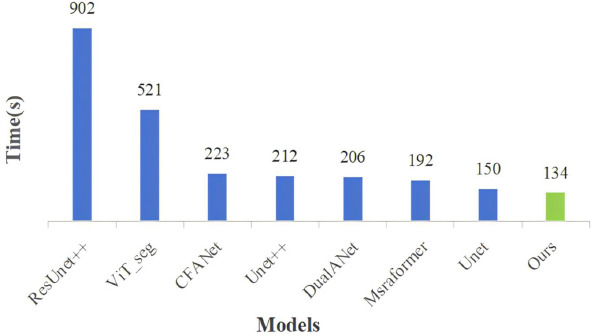
Comparison of the computational speed for different networks.

**Fig. (6):**
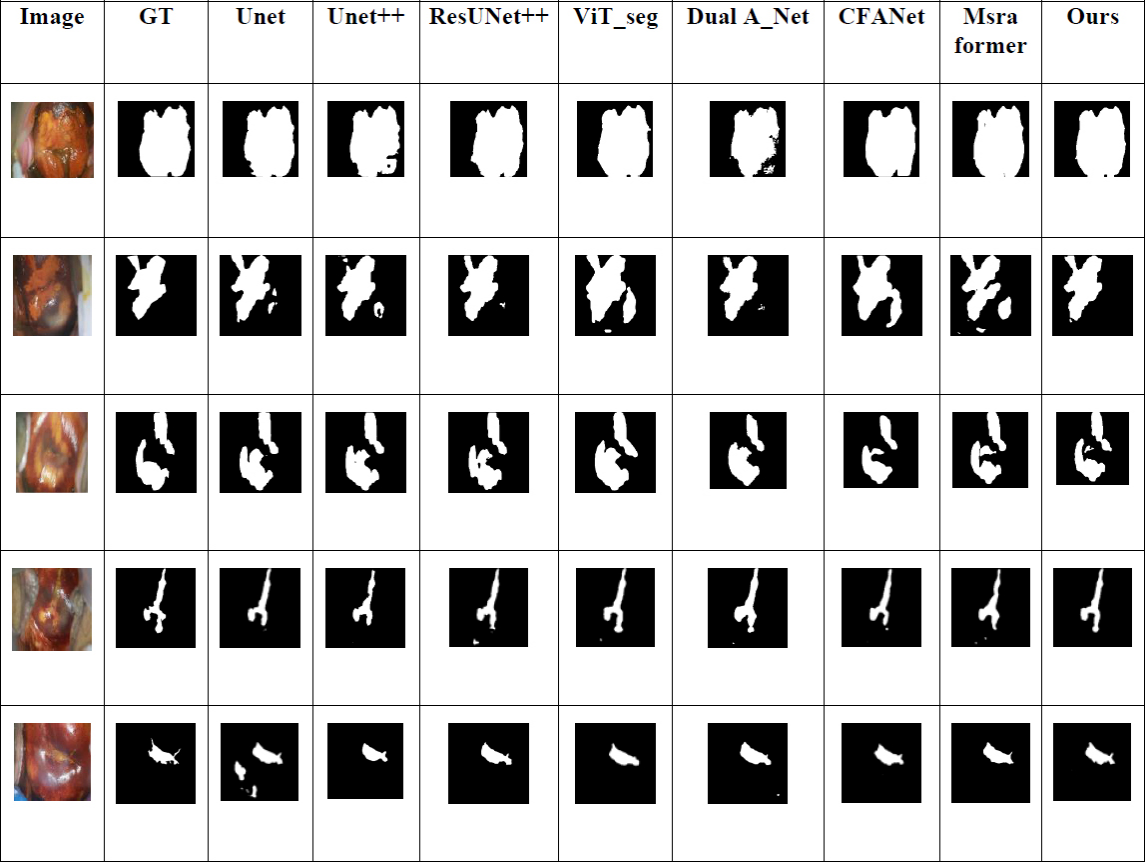
Visualization of cervical cancer image segmentation results obtained by different methods.

**Table 1:** Performance comparison of different segmentation methods on the test dataset used in the paper.

**Methods**	**Recall**	**Specificity**	**Precision**	**F1**	**F2**	**Acc_overcall**	**mIoU**
**Unet**	0.815±0.005	0.965±0.001	0.823±0.004	0.785±0.003	0.791±0.004	0.9458±0.001	0.815±0.002
**Unet++**	0.814±0.002	0.962±0.004	0.804±0.013	0.774±0.012	0.786±0.007	0.943±0.003	0.806±0.008
**ResUNet++**	0.813±0.007	0.959±0.003	0.796±0.011	0.768±0.003	0.783±0.003	0.941±0.002	0.800±0.003
**ViT_seg**	0.853±0.026	0.952±0.008	0.777±0.031	0.779±0.005	0.811±0.011	0.943±0.004	0.805±0.007
**Dual A_Net**	0.741±0.052	0.964±0.010	0.822±0.035	0.727±0.025	0.724±0.038	0.936±0.002	0.776±0.012
**CFANet**	0.784±0.005	0.953±0.004	0.781±0.016	0.737±0.010	0.752±0.007	0.933±0.003	0.636±0.012
**Msraformer**	0.866±0.012	0.954±0.003	0.786±0.011	0.794±0.001	0.822±0.006	0.944±0.002	0.816±0.001
**Ours**	**0.846±0.003**	**0.966±0.001**	**0.836±0.009**	**0.802±0.005**	**0.816±0.004**	**0.949±0.002**	**0.825±0.001**

**Table 2:** Ablation study of different modules.

**Model Configuration**	**Recall**	**Specificity**	**Precision**	**F1**	**F2**	**Acc_** **overcall**	**mIoU**
Backbone (32× Downsampling)	77.67±0.660	95.68±0.073	77.74±0.568	74.29±0.055	75.41±0.278	93.89±0.015	78.25±0.018
Backbone (16× Downsampling)	80.99±0.118	96.46±0.047	80.95±0.247	78.10±0.058	78.88±0.006	94.59±0.019	80.91±0.032
Backbone (16× Downsampling) +MSA	81.77±1.805	96.66±0.084	80.98±0.988	78.72±0.033	79.61±0.616	94.79±0.009	81.33±0.022
Backbone (16× Downsampling) +MSA+BLEU	**84.61±0.028**	**96.69±0.043**	**83.66±0.233**	**80.26±0.031**	**81.67±0.025**	**94.99±0.001**	**82.57±0.011**

**Table 3:** Impact of adaptive parameters in the upsampling module on results.

**Adaptive Parameter**	**Recall**	**Specificity**	**Precision**	**F1**	**F2**	**Acc_** **overcall**	**mIoU**
*α* = 0.7, *β* = 0.2, *γ* = 1.2	83.22±0.026	96.44±0.024	82.51±0.068	79.73±0.143	82.73±0.081	94.88±0.020	82.16±0.081
*α* = 0.65, *β* = 0.1, *γ* = 1.3	83.18±0.441	96.59±0.010	82.54±0.072	79.78±0.295	80.79±0.466	94.92±0.016	82.22±0.087
*α* = 0.6, *β* = 0.1, *γ* = 1.2	**84.61±0.028**	**96.69±0.043**	**83.66±0.233**	**80.26±0.031**	**81.67±0.025**	**94.99±0.001**	**82.57±0.011**

## Data Availability

The data supporting the findings of the article is available in the EVXV repository at https://github.com/Li-Shuhui/ EVXV/tree/master.

## LIST OF ABBREVIATIONS

CNNs: Convolutional Neural Networks
mIoU: mean Intersection over Union
ViT: Vision Transformer
MBLEformer: Multi-Scale Bidirectional Lesion Enhancement Transformer
MSA: Multi-scale attention
